# Accuracy of the Zika IgM Antibody Capture Enzyme-Linked Immunosorbent Assay from the Centers for Disease Control and Prevention (CDC Zika MAC-ELISA) for Diagnosis of Zika Virus Infection

**DOI:** 10.3390/diagnostics10100835

**Published:** 2020-10-18

**Authors:** Moyra Machado Portilho, Laise de Moraes, Mariana Kikuti, Leile Camila Jacob Nascimento, Mitermayer Galvão Reis, Viviane Sampaio Boaventura, Ricardo Khouri, Guilherme Sousa Ribeiro

**Affiliations:** 1 Instituto Gonçalo Moniz, Fundação Oswaldo Cruz, Salvador, BA 40296-710, Brazil; moyra.portilho@hotmail.com (M.M.P.); laisepaixao@live.com (L.d.M.); marianakikuti@gmail.com (M.K.); camila.ufba@yahoo.com.br (L.C.J.N.); mitermayer.reis@fiocruz.br (M.G.R.); vsboaventura@gmail.com (V.S.B.); ricardo_khouri@hotmail.com (R.K.); 2 Faculdade de Medicina, Universidade Federal da Bahia, Salvador, BA 40110-100, Brazil; 3 Instituto de Saúde Coletiva, Universidade Federal da Bahia, Salvador, BA 40110-040, Brazil; 4 Department of Epidemiology of Microbial Diseases, Yale School of Public Health, New Haven, CT 06520, USA

**Keywords:** Zika virus, diagnostic accuracy, sensitivity and specificity, enzyme-linked immunosorbent assay, CDC Zika MAC-ELISA

## Abstract

Serological diagnosis of Zika virus (ZIKV) infection is challenging because of antigenic cross-reactivity with dengue virus (DENV). This study evaluated the accuracy of the Zika IgM antibody capture enzyme-linked immunosorbent assay (CDC Zika IgM MAC-ELISA) in differentiating between ZIKV and DENV infections. To determine sensitivity, we used acute- and convalescent-phase sera from 21 patients with RT-PCR-confirmed ZIKV infection. To determine specificity, we used acute- and convalescent-phase sera from 60 RT-PCR-confirmed dengue cases and sera from 23 blood donors. During the acute-phase of the illness, the assay presented a sensitivity of 12.5% (2/16) for samples collected 0–4 days post symptoms onset (DPSO), and of 75.0% (3/4) for samples collected 5–9 DPSO. During the convalescent-phase of the illness, the test sensitivity was 90.9% (10/11), 100% (2/2), and 0% (0/2) for samples obtained 12–102, 258–260, and 722–727 DPSO, respectively. Specificity for acute- and convalescent-phase samples from RT-PCR-confirmed dengue cases was 100% and 93.2%, respectively. Specificity for blood donor samples was 100%. The assay is an accurate method for Zika serological diagnosis and proved to be reliable for use during surveillance and outbreak investigations in settings where ZIKV and DENV cocirculate.

## 1. Introduction

Zika virus (ZIKV) is a mosquito-borne flavivirus that belongs to the Flaviviridae Family. Although the majority of infections are asymptomatic or produce mild and self-limited clinical manifestations, such as rash, low-grade fever, and arthralgia, it can lead to severe neurological complications, exemplified by the Guillain-Barré syndrome and the congenital Zika syndrome [1,2,3].

Diagnosis of Zika virus (ZIKV) infection is based on clinical, epidemiological, and laboratorial data. Viral RNA can be detected in the first 5–7 days following the onset of symptoms, and, after this period, IgM antibodies may be detected by serological assays [4,5]. However, ZIKV has antigenic similarities with other flaviviruses, especially dengue virus (DENV), which can result in antibody cross-reactivity between the two [6]. As ZIKV and DENV share similar transmission determinants [7,8], serological diagnosis of ZIKV infection is challenging in areas where DENV is endemic.

Studies evaluating the performance of serological assays for ZIKV IgM detection are needed to help clinicians, surveillance personnel, and public health authorities to determine which assays should be used and how to interpret the tests results in order to guide proper clinical management, as well as prevention and control measures. However, few studies have used paired samples to determine the capacity of serological assays to differentiate between ZIKV and DENV infection in settings where both viruses cocirculate. This study aims to evaluate the accuracy of the Zika IgM antibody capture enzyme-linked immunosorbent assay from the Center for Disease Control and Prevention (CDC Zika MAC-ELISA) using a panel of sera samples from febrile patients with RT-PCR-confirmed Zika and dengue, and blood donors, from Bahia, Brazil. In addition, our study provides insights into the kinetics of IgM immune response against ZIKV over time, which help optimize the timing of test use.

## 2. Materials and Methods

### 2.1. Study Design and Sample Selection

To evaluate the sensitivity of the CDC Zika MAC-ELISA, we used a panel of acute- and convalescent-phase sera from 21 febrile patients with RT-PCR-confirmed ZIKV infection. To evaluate the test specificity, we used acute- and convalescent-phase sera from 60 RT-PCR-confirmed dengue patients and sera from 23 blood donors collected prior to the Zika epidemic in the region.

Acute- and convalescent-phase sera from 14 of the 21 RT-PCR-confirmed Zika patients were obtained from a long-term acute febrile illness (AFI) enhanced surveillance study that aimed to detect arboviral infections in a public emergency health unit of Salvador (2.9 million pop.), the capital of the Bahia state, Brazil. Details on the surveillance protocol were described previously [7,9,10]. Briefly, self-reported data on clinical characteristics, including days of symptoms, and an acute-phase blood sample were collected at enrolment and, whenever possible, a convalescent-phase sample was collected ≥10 days later. We were able to obtain a convalescent-phase sample for 8 of the 14 Zika cases. Of note, all the 14 Zika cases were detected between May and July 2015, the period in which ZIKV transmission in Salvador peaked [7,11].

Acute- and convalescent-phase sera from other 7 RT-PCR-confirmed Zika patients were collected during the investigation of a ZIKV outbreak in Campo Formoso, one of the 30 most populous cities from Bahia (~71,000 pop.), in April 2016. These cases underwent ZIKV RT-PCR diagnosis based on the presence of fever and/or maculopapular rash, associated with myalgia, arthralgia, pruritus, headache, or retro-orbital pain, that initiated ≤10 days before the day of interview. Whenever possible, convalescent-phase samples from these patients were collected repeatedly, at different follow-up times. Of these 7 Zika cases, four had two convalescent-phase samples collected and three had only one sample collected, totaling 11 convalescent-phase samples available from this group.

We evaluated the CDC Zika MAC-ELISA specificity in a group of 60 dengue cases selected among the RT-PCR-confirmed dengue cases detected during the AFI enhanced surveillance study previously described. These 60 dengue cases were the first 20 cases of DENV-1, DENV-2, and DENV-4 detected during the AFI surveillance study for which paired acute- and convalescent sera were available. Samples from these dengue cases were collected between February 2009 and May 2011, before the estimated introduction of ZIKV in Brazil, i.e., between late 2012 and early 2013 [12,13,14]. We also tested the acute-phase sera available for 47 of these patients by ZIKV RT-PCR [15] to discard a concomitant ZIKV infection; all of them tested negative. DENV-3 patients were not included in this assessment due to the low number of DENV-3 cases identified by our surveillance prior to ZIKV introduction in Brazil.

To assess the test specificity, we also included sera samples from 23 blood donors obtained in December 2013, prior the first ZIKV case detection in Bahia [11,16]. All blood donors underwent regular health screening for donation, which included absence of symptoms, such as fever, in the 15 days prior to the donation.

All blood samples were kept refrigerated until centrifugation, and the obtained sera were stored at −20 °C and −80 °C for serological and molecular testing, respectively.

This study was approved by The Research Ethics Committee of Instituto Gonçalo Moniz, Fundação Oswaldo Cruz (number 3.363.703; CAAE: 55904616.4.0000.0040; 3 June 2019) and School of Medicine, University Federal of Bahia (UFBA) (number 1.657.324; CAAE 56910516.3.0000.5577; 1 August 2016). All adult subjects provided written informed consent and participants <18 years of age who were able to read provided written assent following written consent from their parent or guardian.

### 2.2. Laboratorial Tests

All acute-phase samples underwent RNA extraction using the Maxwell^®^ 16 Total RNA Purification kit (Promega, WI, USA) or QIAmp^®^ Viral RNA Mini kit commercial kit (Qiagen, Hilden, Germany) according to manufacturer’s instructions. We then performed RT-PCR on each extract product using specific oligonucleotides to amplify DENV [17] and ZIKV [15] separately, and/or multiplex qRT-PCR for ZIKV and DENV [18]. We also tested acute-phase sera samples with DENV NS1-, IgG- and IgM-ELISA (Abbott, Santa Clara, CA, USA; former Panbio Diagnostics, Brisbane, Australia) according to manufacturer’s instructions.

All acute- and convalescence-phase study samples were tested for the presence of IgM anti-ZIKV antibodies using the CDC Zika MAC-ELISA protocol (NIAID-NIH BEI Resources Repository, catalog no. NR-50449) [4]. Briefly, 96-well high-affinity microtiter plates (Nunc MaxiSorp flat-bottom 96 well plates) were coated with goat anti-human IgM antibody diluted at 1:1000 (Kirkegaard and Perry Laboratories, Gaithersburg, MD, USA, catalog #01-10-03) and incubated at 4 °C overnight. The next day, plates were washed with phosphate buffer with 0.05% tween 20 (PBS-T), and nonspecific binding sites were blocked with PBS-T containing 5% nonfat dry milk for 1h at room temperature. Meanwhile, serum samples were incubated at 56 °C for two hours for arboviral inactivation. Plates were washed with PBS-T, and each inactivated serum samples and IgM anti-ZIKV positive and negative controls provided by CDC and diluted at 1:400 were added to four wells and incubated at 37 °C for 1 h. Plates were washed with PBS-T, and each Zika Vero E6 and Normal Vero E6 cell culture antigen were splitted in two of the four wells of patients′ and controls′ samples, and incubated at 4 °C overnight. The next day, plates were washed and horseradish peroxidase (HRP)-conjugated monoclonal antibody 6B6C-1 (Hennessy Research, Lenexa, KS, USA, catalog #DC153-100) diluted at 1:5000 was added to the plates and incubated at 37 °C for 1h. Plates were washed and blue tetramethylbenzidine (TMB) substrate was added and incubated protected from light, at room temperature (20–25 °C) for 10 min. The enzyme/substrate reaction was stopped by adding the stop solution (H_2_SO_4_) and incubated at room temperature for 5 min before reading using an optical density microplate reader (FilterMax F3, Molecular Devices, Sunnyvale, CA, USA) and SoftMax Pro 6.2 Software.

Results of the CDC Zika MAC-ELISA were determined by calculating the ratio of the optical density mean (OD) of the wells containing Zika Vero E6 cell culture antigen (P) divided by the normal Vero E6 OD mean (N) for each patient and control sample. Ratio values obtained for each patient were classified as negative when P/N < 2, inconclusive when P/N ranged from 2 to <3, and positive when P/N ≥ 3. Samples with inconclusive results were retested and the result of the repetition was considered final.

The samples from the RT-PCR-positive Zika cases from Campo Formoso were the first to be tested by the CDC Zika MAC-ELISA, and this testing was not blinded. However, all the remaining samples, including the acute- and convalescent-phase samples from Zika and dengue cases from Salvador and the blood donor samples, were randomly numbered for deidentification and blindness in the CDC Zika MAC ELISA tests.

### 2.3. Data Analysis

We used absolute and relative frequency or median and interquartile range (IQR) to characterize the RT-PCR-positive Zika cases (overall and by place of residency) and the RT-PCR-positive dengue cases, according to demographics and clinical manifestations. We calculated sensitivities and 95% confidence intervals (95% CI) amongst RT-PCR-positive Zika cases and specificities, and 95% CI amongst RT-PCR-positive dengue cases, as well as amongst blood donors. Sensitivity was calculated according to timing of serum sample collection, to determine the presence of antibody kinetics during infection. Thus, acute-phase samples were classified as early acute-phase (collected 0–4 days post symptoms onset (DPSO)), and late acute-phase (5–9 DPSO); convalescent-phase samples were classified into three periods: early convalescent-phase (12–102 DPSO), intermediate convalescent-phase (258–260 DPSO), and late convalescent-phase (722–727 DPSO). The CDC Zika MAC-ELISA specificity was also stratified by the infecting DENV serotype and by type of DENV infection (primary versus secondary). We excluded from the accuracy analysis samples with a final inconclusive MAC-ELISA result.

In order to obtain information about the kinetics of the Zika IgM antibodies detected by the CDC Zika MAC-ELISA, we graphically plotted the median and IQR of the P/N ratio values obtained for the Zika cases at the different time points of sample collection (early acute, late acute, early convalescent, intermediate convalescent, and late convalescent). We also plotted the same graph with individual ratios per Zika positive case.

## 3. Results

### 3.1. Participant Characteristics

Of the 21 RT-PCR-positive Zika cases, 61.9% were female and the median age was 24 years old (Table 1). Of the 60 RT-PCR-positive DENV cases, 48.3% were female and the median age was 18 years old. Of the 23 blood donors, 30.4% were female and the median age was 32 years old.

RT-PCR-positive Zika and dengue cases were similar regarding the frequency of headache (95.2% vs. 96.7%, respectively) and myalgia (80.9% vs. 76.7%), but Zika cases more frequently had rash (76.2% vs. 10.0%), retro-orbital pain (76.2% vs. 56.7%), and arthralgia (66.7% vs. 46.7%), compared to dengue cases (Table 1). Zika cases from Salvador were not particularly different from those from Campo Formoso regarding clinical signs, except for vomiting, which was reported among 14.3% of the Salvador cases and 71.4% of the Campo Formoso cases; 20.0% of the dengue cases reported vomiting.

Two RT-PCR-positive Zika cases were also RT-PCR-positive for DENV. One of them had detectable DENV IgM and IgG antibodies in its acute-phase sample, and the other only had detectable IgG. Of note, previous DENV infection, determined by a positive DENV IgG ELISA result in the acute-phase sample (indicating a secondary dengue infection), was observed for 15 (75.0%) of the 20 Zika cases with available results, for 78.3% the RT-PCR-positive dengue cases, and for 87.0% of the blood donors.

### 3.2. CDC Zika MAC-ELISA Performance

At the initial round of tests, 173 (94.5%) of the 183 samples evaluated presented a valid result. After retesting 9 of the 10 samples that presented an inconclusive result (one did not have sufficient volume for retesting), the total number of samples presenting a valid result increased to 176 (96.7%) and included 20 acute- and 15 convalescent-phase samples from Zika cases, 59 acute- and 59 convalescent-phase samples from dengue cases, and all the 23 samples from blood donors. The valid results were used to evaluate the CDC Zika MAC-ELISA accuracy.

The overall CDC Zika IgM MAC-ELISA sensitivity on acute-phase samples was 25.0%, but it increased from 12.5% on early acute-phase samples to 75.0% on late acute-phase samples (Table 2). The test sensitivity on convalescent-phase samples was 90.9%, 100% and 0.0% at early, intermediate, and late convalescence, respectively. For the unique ZIKV RT-PCR-positive case that had DENV IgM detected in the acute-phase sample (the case with a concomitant DENV infection), the CDC Zika IgM-MAC-ELISA performed in the same sample was also positive. The sensitivity was also related with the qRT-PCR cycle threshold (CT) at which the samples were defined as positive. Acute-phase samples with higher (≥34) CT values were more frequently positive by the CDC Zika IgM MAC-ELISA than samples with lower (<34) CT values (42.9% vs. 9.1%, respectively).

**Figure 1:**
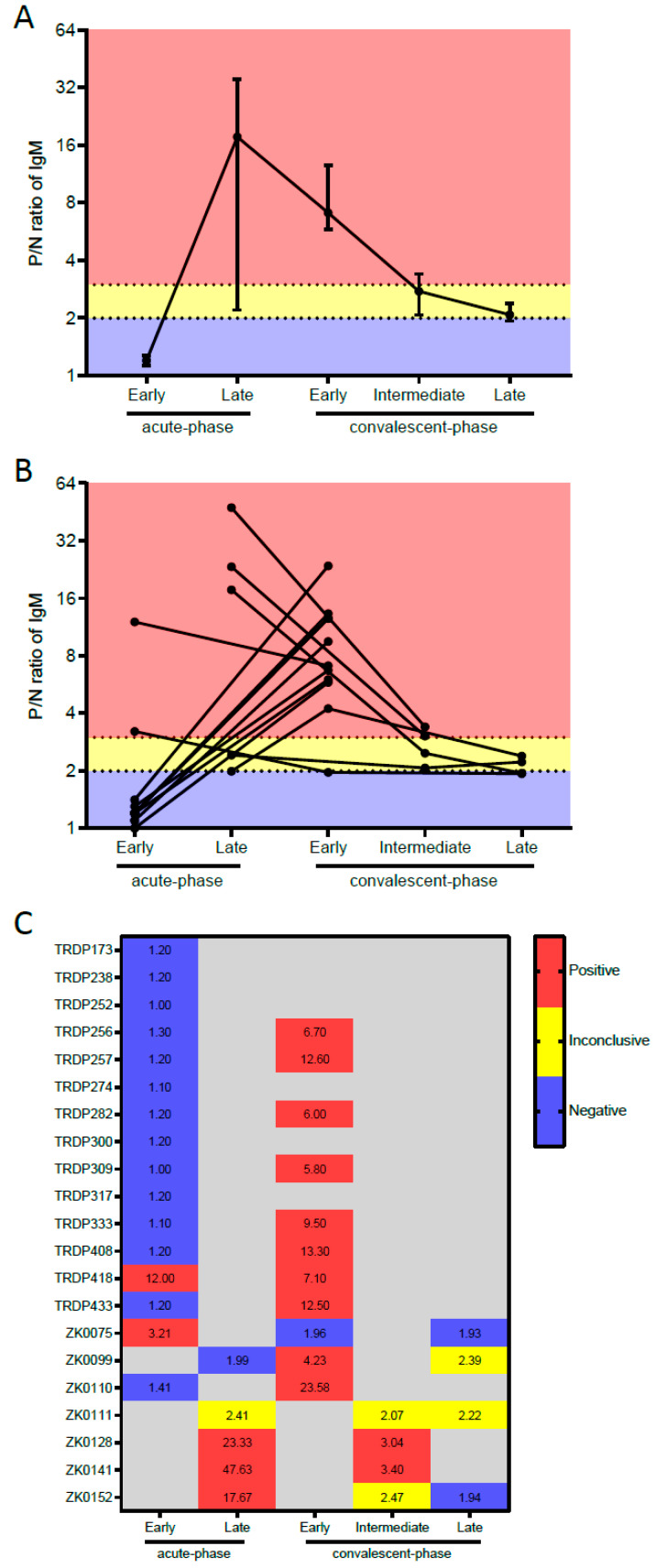
Kinetics of IgM detection against ZIKV over time. Positive/negative (P/N) ratio values obtained by ZIKAV MAC-ELISA-CDC from serum samples from ZIKAV-infected patients originally diagnosed by qPCR Trioplex-CDC at different time points (Acute phase, Early convalescent phase, Intermediate convalescent phase, and Late convalescent phase). (**A**) Line plot with superimposed symbols at median with interquartile range, (**B**) Dot plot with before/after lines per patient and (**C**) Heatmap indicating the P/N ratio values of IgM for each ZIKV-infected patient serum samples. Legend: blue (negative—P/N < 2), yellow (inconclusive—2 < P/N < 3), red (positive—P/N ≥ 3) and grey (missing sample due to different cohort design).

The kinetics of the Zika IgM antibodies detected by the CDC Zika MAC-ELISA over time revealed a rapid augmentation in IgM antibody response from the early to the late acute-phase of illness (Figure 1). Then, the IgM antibody levels decreased, but remained high enough to be detected by the test as positive for all except one of the 11 patients with an early convalescent-phase sample collected (between 12 and 61 DPSO). The IgM levels continued to decrease over time, and only two of the four samples collected at an intermediate convalescent-phase remained positive (the other two presented inconclusive results). At a late convalescent-phase, none of the four tested samples had sufficient antibodies to be considered positive (two were negative and two were inconclusive).

The test specificity was 100% and 93.2% on acute- and convalescent-phase samples from dengue cases, respectively, and 100% for blood donor samples (Table 2). The specificity of the convalescent-phase samples from dengue cases did not vary substantially according to the DENV serotype (90.0% for DENV-1, 95.0% for DENV-2, and 94.7% for DENV-4) or the type of infection (92.3% for primary dengue and 93.5% for secondary dengue). Five of the DENV RT-PCR positive samples also presented detectable DENV-IgM in the acute-phase sample, and none of them was positive by the CDC Zika MAC-ELISA.

## 4. Discussion

We evaluated the performance of the CDC Zika IgM-MAC-ELISA for Zika diagnosis, aiming to determine its sensitivity at different sampling time points during the disease course. We also evaluated the test specificity on sera from RT-PCR-positive dengue cases and from blood donors from a dengue endemic setting, which is particularly important given DENV and ZIKV cocirculation, the clinical similarity in the signs and symptoms they cause [7], and the potential for immunological cross-reactions due to the close genetic and antigenic relationship between them [19]. Furthermore, the panel of DENV samples was obtained from well characterized patients regarding clinical and laboratory data, allowing a detailed evaluation to be made of the assay specificity (i.e., according to infecting DENV serotype and presence of DENV antibodies at acute-phase sample collection).

As expected due to the short time elapsed from onset of symptoms, the CDC Zika IgM MAC-ELISA sensitivity for acute-phase samples obtained in the first four days was very poor (12.5%), but it increased after 5 days of illness (75.0%), and was high (90.9%) for samples obtained early during convalescence (12–102 DPSO). It is noteworthy that our findings on the kinetics of ZIKV IgM antibody response, as revealed by the serial sampling of RT-PCR-confirmed Zika patients at different time points, are in agreement with those of other studies that showed that ZIKV IgM antibodies starts to be detected four or five days post infection and maintain detectable levels for at least twelve weeks [5,20].

The test also showed high specificity when applied to the acute- and convalescent-phase sera of RT-PCR-confirmed dengue cases (100.0% and 93.2%, respectively) and to blood donor sera (100.0%). Lower specificity was found in the subgroup of convalescent-phase sera from DENV-1 cases, but it was not substantially different from those observed for DENV-2 and DENV-4 cases. The specificity was very similar for samples of primary and secondary DENV infection cases. Of note, none of the five acute-phase samples that were RT-PCR and IgM positive for DENV returned a positive result in the CDC Zika IgM MAC-ELISA, suggesting that the observed early DENV IgM response did not cross-react on the Zika IgM MAC-ELISA, or was not strong enough to be detected. On the other hand, the unique acute-phase sample from a ZIKV RT-PCR-positive case that was also positive for DENV by RT-PCR and IgM-ELISA also tested positive in the CDC Zika IgM MAC-ELISA. This finding may be due to DENV and ZIKV antibody cross-reaction, but the limited number of samples fulfilling this condition hampers a proper conclusion.

The CDC assay has been evaluated before [21,22,23,24,25]. In general, the evaluations showed that its accuracy is high, but the results were somewhat varied. One study, that, like ours, used specimens from RT-PCR-positive patients to evaluate the test sensitivity at different time points, had similar findings: 34.6% for samples collected at 1–4 DPSO (similar to our early acute-phase group), 74.3% at 5–10 DPSO (similar to our late acute-phase group), and 83% at 11–34 DPSO (similar to our early convalescence-phase group) [21]. However, specificity was not determined. Another study evaluated the CDC Zika IgM MAC-ELISA and the combined commercial Euroimmun anti-Zika Virus IgM and IgG ELISA assays (Euroimmun AG, Lübeck, Germany) against PRNT results and found sensitivities of 100% and 83.3%, respectively, and specificities of 47.1 and 81.2%, respectively [22].

Some studies [23,24] used the CDC Zika IgM MAC-ELISA as the standard method for comparison with commercial ELISAs. Granger and colleagues compared the InBios ZIKV Detect IgM capture ELISA (InBios MAC-ELISA; InBios International, Inc., Seattle, WA, USA) and the Euroimmun anti-Zika Virus IgM ELISA (Euroimmun ELISA; Euroimmun AG, Lübeck, Germany) to the CDC Zika IgM MAC-ELISA. Their overall agreement was 90.7% and 51.9%, respectively, which might suggest that the CDC and the Inbios tests had better performance than the Euroimmun test [23]. Safronetz and colleagues evaluated four Zika IgM ELISA methodologies in comparison to the CDC Zika IgM MAC-ELISA [24]. Although the study did not aim to evaluate the performance of the CDC Zika IgM MAC-ELISA, the data provided allowed us to estimate the CDC MAC-ELISA sensitivity for 30 samples that were PRNT-positive for ZIKV and 10 samples that were RT-PCR-positive for ZIKV (100% and 20.0%, respectively). It also allowed us to estimate the specificity for 10 samples that were PRNT-positive for DENV and for 25 samples that were RT-PCR-negative for ZIKV (0.0% and 100%, respectively). Three of the four evaluated ZIKV IgM ELISAs had low sensitivities against the CDC test, and only the InBios Zika Virus Detect MAC-ELISA showed a performance comparable to the CDC ELISA.

The accuracy of the CDC Zika IgM MAC-ELISA and another MAC-ELISA methodology (CNDR MAC-ELISA, Centro Nacional de Diagnóstico y Referencia (CNDR) of the Nicaraguan Ministry of Health) were also determined against a composite reference that included results from RT-PCR and serological methods for ZIKV, and RT-PCR for DENV in a panel samples from a pediatric cohort [25]. Their sensitivities were 70.1% and 94.5%, and their specificities were 82.8% and 85.6%, respectively. According to the authors, the different performance may be related to the diverse antiflavivirus monoclonal antibodies used in the ELISAs, which were less specific in the CDC Zika IgM MAC-ELISA than in the CNDR MAC-ELISA.

Of note, we have previously used the same panel of samples (except for the RT-PCR-positive ZIKV samples from Campo Formoso) to evaluate the Euroimmun anti-Zika Virus IgM ELISA (Euroimmun ELISA; Euroimmun AG, Lübeck, Germany) [26], which allowed a direct test-to-test comparison to be made between the results of this and the previously published study. The sensitivity for the CDC and the Euroimmun tests on the same acute-phase samples from RT-PCR-confirmed Zika patients were 7.1% (1/14) and 0.0% (0/14), respectively (McNemar *p* value: 0.48), and on the same early convalescent-phase samples were 100.0% (8/8) and 12.5% (1/8) (McNemar *p* value: 0.01), respectively (Table A1 in Appendix A). Both tests had high specificities, but for the convalescent-phase samples of RT-PCR-confirmed Dengue patients, the specificity of the Euroimmun test was nonstatistically higher than the CDC test (98.3% (58/59) vs. 93.2% (55/59), respectively, McNemar *p* value: 0.18). Altogether, the CDC Zika IgM MAC-ELISA was superior to the Euroimmun anti-Zika Virus IgM ELISA.

Although the CDC Zika IgM MAC-ELISA presented satisfactory results, 10 (5.5%) of the initially 183 tested samples presented inconclusive results and needed to be repeated; after retesting 9 of them, 6 (3.3%) remained inconclusive. These 6 samples were from 5 RT-PCR-confirmed Zika cases (1 acute-phase and 4 convalescent-phase samples from intermediate and late periods) and from 1 RT-PCR-confirmed dengue case (acute-phase). The inconclusive results may be due to background reactivity or to the inherent interlaboratory variability associated with the CDC Zika IgM MAC-ELISA preparation, as the assay requires site-specific optimization of the dilutions used for select reagents, such as ZIKV antigen, goat anti-human IgM or conjugated secondary antibody [4]. However, the low frequency of invalid results and the good test performance indicated that the CDC Zika MAC-ELISA is reproducible and reliable.

Despite its high accuracy, the time of execution of CDC Zika IgM MAC-ELISA is an inherent limitation. As an in-house method, it requires covering the plate with IgM antibodies and overnight incubation, taking a total of two or three days to obtain the test results. Thus, in the context of large outbreaks, when several thousand samples need to be tested, this assay may not be practical, and standardization and evaluation of ready-to-use commercial methods for ZIKV serological diagnosis remain necessary.

This study has some limitations. It has been suggested that the PRNT should be used as the reference method for confirmation of the presence of anti-Zika antibodies [4]. Although we lacked PRNT results, we classified positive and negative samples for ZIKV and DENV according to patient RT-PCR results during the acute-phase of disease, which ensured a more accurate distinction between these flaviviruses compared to serologic diagnosis. Second, although we increased the number of samples from RT-PCR-confirmed Zika patients in comparison to our previous study evaluating the Euroimmun IgM ELISA [26], we still had a relatively small number of samples from Zika cases, and might lack power for comparisons, particularly when stratifying them according to the time of sample collection. Third, we did not evaluate the test specificity of control samples obtained after introduction of ZIKV in Brazil. Theoretically, prior ZIKV exposure may modify the likelihood of false-positive results. In addition, the immune response of individuals who had sequential flavivirus infections in the past (i.e., DENV infection following ZIKV infection or ZIKV infection following DENV infection) may differ from that of naïve individuals, possibly altering the performance of ZIKV serological tests. Further studies evaluating the accuracy of flavivirus serological tests may benefit from the inclusion of control samples obtained in the period following ZIKV introduction.

## 5. Conclusions

Given the cross-immunity between ZIKV and DENV infections, development and validation of serological tests for Zika diagnosis need to specifically consider the possibility of false positive reactions when the test is used on dengue patients or in patients with prior DENV infection. Our study has the merit of evaluating a Zika IgM MAC-ELISA against a panel of paired samples obtained from clinically well-characterized patients with RT-PCR-confirmed Zika and dengue. Dengue patients comprised infections in three of the four DENV serotypes, and nearly 80% of them had a secondary infection. Thus, our study mimicked a scenario of ZIKV diagnosis in a region of endemic DENV transmission. We found that the CDC Zika IgM MAC-ELISA assay presented an optimal sensitivity for diagnosis of ZIKV infection when applied to samples collected at the early and intermediate convalescent phases. Furthermore, the test also had a high specificity, even when used on convalescent-phase samples of RT-PCR-confirmed dengue cases and on samples of blood donors with serological evidence of prior DENV infection. Thus, it is an accurate method for Zika diagnosis during surveillance and outbreak investigations in dengue endemic regions.

## Figures and Tables

**Table 1:** Demographic, clinical and laboratorial characteristics, and serum sample availability for RT-PCR-confirmed Zika (*n* = 21) and dengue (*n* = 60) cases.

Characteristics	Zika Cases			Dengue Cases from Salvador			
	from Salvador (*n* = 14)	from Campo Formoso (*n* = 7)	Total (*n* = 21)	DENV-1 (*n* = 20)	DENV-2 (*n* = 20)	DENV-4 (*n* = 20)	Total (*n* = 60)
	Number (%) or median (interquartile range)						
Demographic							
Female	8 (57.1)	5 (71.4)	13 (61.9)	9 (45.0)	10 (50.0)	10 (50.0)	29 (48.3)
Age	22.5 (15–41)	24 (10–34)	24 (14–38)	20 (10.5–37)	13.5 (9.5–22)	22.5 (10.5–34.5)	18 (10–34)
Clinical manifestation							
Fever	14 (100)	7 (100)	21 (100)	20 (100)	20 (100)	20 (100)	60 (100)
Headache	13 (92.9)	7 (100)	20 (95.2)	19 (95.0)	19 (95.0)	20 (100)	58 (96.7)
Myalgia	12 (85.7)	5 (71.4)	17 (80.9)	18 (90.0)	14 (70.0)	14 (70.0)	46 (76.7)
Rash	10 (71.4)	6 (85.7)	16 (76.2)	1 (5.0)	5 (25.0)	0	6 (10.0)
Pruritus	10 (71.4)	6 (85.7)	16 (76.2)	NA	NA	NA	NA
Retro-orbital pain	10 (71.4)	6 (85.7)	16 (76.2)	14 (70.0)	9 (45.0)	11 (55.0)	34 (56.7)
Arthralgia	8 (57.1)	6 (85.7)	14 (66.7)	10 (50.0)	8 (40.0)	10 (50.0)	28 (46.7)
Vomiting	2 (14.3)	5 (71.4)	7 (33.3)	6 (30.0)	2 (10.0)	4 (20.0)	12 (20.0)
Blood sample collection							
Acute-phase sample							
Early (0–4 DPSO)	14 (100)	2 (28.6)	16 (76.2)	19 (95.0)	20 (100)	20 (100)	59 (98.3)
Late (5–9 DPSO)	0	5 (71.4)	5 (23.8)	1 (5.0)	0	0	1 (1.7)
Convalescent-phase sample ^a^							
Early (12–102 DPSO)	8 (57.1)	3 (42.9)	11 (52.4)	20 (100)	20 (100)	20 (100)	60 (100)
Intermediate (258–260 DPSO)	0	4 (57.1)	4 (57.1)	0	0	0	0
Late (722–727 DPSO)	0	4 (57.1)	4 (57.1)	0	0	0	0
ZIKV diagnosis							
RT-PCR positivity	14 (100)	NA	14 (100)	0	0	0	0 ^b^
qRT-PCR positivity	12 (85.7)	7 (100)	19 (90.5)	NA	NA	NA	NA
Ct value on qRT-PCR	32.3 (30.0–36.7)	34.3 (26.3–36.8)	33.6 (30.0–36.8)	NA	NA	NA	NA
DENV diagnosis							
RT-PCR positivity ^c^	1 (7.1)	1 (14.3)	2 (9.5)	20 (100)	20 (100)	20 (100)	60 (100)
NS1-ELISA positivity	0	0	0	15 (75.0)	6 (30.0)	15 (75.0)	36 (60.0)
IgM-ELISA positivity ^d^	1 (7.1)	0	1 (4.8)	4 (20.0)	0	2 (10.0)	6 (10.0)
Prior DENV infection ^e^	11 (84.6)	4 (57.1)	15 (75.0)	13 (65.0)	14 (70.0)	20 (100)	47 (78.3)

NA, Not available or applicable; DPSO, days post symptoms onset; ZIKV, Zika virus; DENV, dengue virus; Ct, cycle threshold; NS1, Nonstructural 1 protein. ^a^ Convalescent-phase samples of Zika patients were not available for 6 cases from Salvador; ^b^ Of the 60 RT-PCR-confirmed dengue cases, 47 had an acute-phase sample available for ZIKV RT-PCR testing, all of which had a negative result; ^c^ Two RT-PCR-confirmed Zika cases also presented a positive result for DENV RT-PCR; ^d^ IgM-ELISA performed on the acute-phase samples. One RT-PCR-confirmed Zika case (which had a DENV co-infection detected by RT-PCR) also presented a positive result for DENV IgM; ^e^ Prior dengue infection was determined by a positive result in the DENV IgG-ELISA performed in the acute-phase serum sample. One ZIKV RT-PCR positive sample from Salvador did not have DENV IgG-ELISA result.

**Table 2:** Performance of the CDC ZIKV IgM-MAC-ELISA assay.

Performance according to the Type of Serum Samples	Tested Samples	ZIKV IgM-Mac-ELISA Result		% (95% CI)
		Pos.	Neg.	
Sensitivity against RT-PCR-confirmed Zika cases				
Acute-phase samples				
Overall ^a^	20	5	15	25.0 (8.7–49.1)
Early (0–4 DPSO)	16	2	14	12.5 (1.5–38.3)
Late (5–9 DPSO) ^a^	4	3	1	75.0 (19.4–99.4)
CT value on qRT-PCR <34 ^b^	11	1	10	9.1 (0.2–41.3)
CT value on qRT-PCR ≥34 ^a,b^	7	3	4	42.9 (9.9–81.6)
Convalescent-phase samples				
Early (12–102 DPSO)	11	10	1	90.9 (58.7–99.8)
Intermediate (258–260 DPSO) ^c^	2	2	0	100 (15.8–100)
Late convalescent-phase samples (722–727 DPSO) ^d^	2	0	2	0.0 (0.0–84.2)
CT value on qRT-PCR <34 ^b,e^	5	5	0	100 (47.9–100)
CT value on qRT-PCR ≥34 ^b,e^	4	3	1	75.0 (19.4–99.4)
Specificity against RT-PCR-confirmed dengue cases				
Acute-phase samples				
Overall ^f^	59	0	59	100 (93.9–100)
DENV-1 cases ^f^	19	0	19	100 (82.3–100)
DENV-2 cases	20	0	20	100 (83.2–100)
DENV-4 cases	20	0	20	100 (83.2–100)
Primary DENV infection	13	0	13	100 (75.3–100)
Secondary DENV infection ^f^	46	0	46	100 (92.3–100)
Convalescent-phase samples				
Overall ^g^	59	4	55	93.2 (83.5–98.1)
DENV-1 cases	20	2	18	90.0 (68.3–98.8)
DENV-2 cases	20	1	19	95.0 (75.1–99.9)
DENV-4 cases ^g^	19	1	18	94.7 (74.0–99.9)
Primary DENV infection	13	1	12	92.3 (64.0–99.8)
Secondary DENV infection ^g^	46	3	43	93.5 (82.1–98.6)
Specificity against blood donors’ samples	23	0	23	100 (85.2–100)

DPSO, days post symptoms onset; CT, cycle threshold; ZIKV, Zika virus; DENV, dengue virus. ^a^ Sensitivity assessment excluded one late acute-phase sample from Campo Formoso with inconclusive MAC-ELISA result; ^b^ Sensitivity assessment based on the qRT-PCR CT measured in the acute-phase samples. This analysis excluded two patients whose acute-phase sample was not evaluated by qRT-PCR; ^c^ Sensitivity assessment excluded two intermediate convalescent-phase samples with inconclusive MAC-ELISA results; ^d^ Sensitivity assessment excluded two late convalescent-phase samples with inconclusive MAC-ELISA result; ^e^ Sensitivity assessment measured for the early convalescent-phase samples; ^f^ Specificity assessment excluded one acute-phase sample with inconclusive MAC-ELISA result; ^g^ Specificity assessment excluded one convalescent-phase sample with inconclusive MAC-ELISA result.

## Appendix A

**Table A1:** Evaluation of the accuracy of the CDC IgM Zika MAC-ELISA compared to the Euroimmun anti-Zika Virus IgM ELISA on the same set of acute- and convalescent-phase samples from RT-PCR-confirmed Zika patients, RT-PCR-confirmed dengue patients, and of blood donor samples from Salvador, Brazil.

	Test Result		% (95% CI)	McNemar *p* Value
	Pos.	Neg.		
Sensitivity				
Acute-phase samples				
CDC ZIKV IgM MAC-ELISA	1	13	7.1 (0.2–33.9)	0.48
Euroimmun anti-ZikaVirusIgM ELISA	0	14	0.0 (0.0–23.2)	
Convalescent-phase samples				
CDC ZIKV IgM MAC-ELISA	8	0	100 (63.1–100)	0.01
Euroimmun anti-ZikaVirusIgM ELISA	1	7	12.5 (0.3–52.6)	
Specificity against RT-PCR-confirmed dengue cases				
Acute-phase samples				
CDC ZIKV IgM MAC-ELISA ^a^	0	59	100 (93.9–100)	1.00 ^c^
Euroimmun anti-ZikaVirusIgMELISA ^b^	0	58	100 (93.8–100)	
Convalescent-phase samples				
CDC ZIKV IgM MAC-ELISA ^d^	4	55	93.2 (83.5–98.1)	0.18 ^f^
Euroimmun anti-ZikaVirusIgMELISA ^e^	1	58	98.3 (90.9–100)	
Specificity against blood donors’ samples				
CDC ZIKV IgM MAC-ELISA	0	23	100 (85.2–100)	1.00
Euroimmun anti-ZikaVirusIgM ELISA	0	23	100 (85.2–100)	

^a^ Specificity assessment excluded one sample with inconclusive MAC-ELISA result; ^b^ Specificity assessment excluded two sample with no results by the Euroimmun assay; ^c^ Comparison of the two tests’ specificities by the McNemar test excluded one sample with inconclusive MAC-ELISA result and two samples with no result by the Euroimmun assay (*N* = 57); ^d^ Specificity assessment excluded one sample with inconclusive MAC-ELISA result; ^e^ Specificity assessment excluded one sample with no result by the Euroimmun assay; ^f^ Comparison of the two tests’ specificities by the McNemar test excluded one sample with inconclusive MAC-ELISA result and one sample with no result by the Euroimmun assay (*N* = 58).
